# *In-silico* techniques to inform and improve the personalized prescription of shoe insoles

**DOI:** 10.3389/fbioe.2024.1351403

**Published:** 2024-02-23

**Authors:** Bryce A. Killen, Sam Van Rossom, Fien Burg, Jos Vander Sloten, Ilse Jonkers

**Affiliations:** ^1^ Human Movement Biomechanics Research Group, Department of Movement Sciences, KU Leuven, Leuven, Belgium; ^2^ Materialise Motion, Materialise, Leuven, Belgium; ^3^ Biomechanics Section, Department of Mechanical Engineering, Faculty of Engineering Sciences, KU Leuven, Heverlee (Leuven), Belgium

**Keywords:** *in-silico*, musculoskeletal, insole ankle foot orthotic, ankle, design methodology

## Abstract

**Background:** Corrective shoe insoles are prescribed for a range of foot deformities and are typically designed based on a subjective assessment limiting personalization and potentially leading to sub optimal treatment outcomes. The incorporation of *in silico* techniques in the design and customization of insoles may improve personalized correction and hence insole efficiency.

**Methods:** We developed an *in silico* workflow for insole design and customization using a combination of measured motion capture, inverse musculoskeletal modelling as well as forward simulation approaches to predict the kinematic response to specific insole designs. The developed workflow was tested on twenty-seven participants containing a combination of healthy participants (7) and patients with flatfoot deformity (20).

**Results:** Average error between measured and simulated kinematics were 4.7 ± 3.1, 4.5 ± 3.1, 2.3 ± 2.3, and 2.3 ± 2.7° for the chopart obliquity, chopart anterior-posterior axis, tarsometatarsal first ray, and tarsometatarsal fifth ray joints respectively.

**Discussion**: The developed workflow offers distinct advantages to previous modeling workflows such as speed of use, use of more accessible data, use of only open-source software, and is highly automated. It provides a solid basis for future work on improving predictive accuracy by adapting the currently implemented insole model and incorporating additional data such as plantar pressure.

## Introduction

The foot-ankle is a highly intricate collection of bones, and joints supported by a complex system of soft tissues including ligaments, tendons, and muscles (Golanó et al., 2010; Merian et al., 2011). This complex structure is matched by the complex function it performs. The foot-ankle complex forms the connection with the ground and is essential for a majority of tasks of daily living, and all locomotion tasks (Matsui et al., 2017). Due to the complexity and its load bearing role, the foot-ankle is highly prone to injury and by extension dysfunction. Injuries to the foot-ankle complex can be acute in nature such as a lateral ligament tear, however chronic conditions may occur due to foot congenital predisposition, ligament overloading and subsequent laxity, inducing chronic pain and ultimately osteoarthritis (Arnold et al., 2019).

Treatment for many of these chronic conditions involves, in combination with conservative physical therapy, prescription of corrective insoles. Such insoles are common treatment for flatfoot deformity (Glassman et al., 2006; Kido et al., 2014; Su et al., 2017) which aim to correct the structural deformity and support the foot. Other common uses are for diabetes mellitus (Paton et al., 2011) whereby insoles are prescribed to alleviate high pressure concentrations under the foot. Despite their widespread use and prescription, the decision-making within the design process for patient-specific insole design is largely reliant on outdated, and subjective approaches (Bacarin et al., 2009; Paton et al., 2011).

The design of insoles is typically reliant predominantly on clinical experience of the podiatrist prescribing the insole. This clinical design-making is often supplemented by limited quantitative measurements such as static footprints and in some rare cases limited single plane x-ray imaging. These measurements and assessments fail to properly consider the function of the foot under dynamic conditions, where the insole should exert its corrective function. Despite this, attempts are being made to assess foot function more objectively through a range of different methods including video-based 2D movement analysis, dynamic plantar pressure measurement and in some cases 3D motion capture. The analysis of the dynamic motion of the numerous joints of the foot-ankle complex can allow for a deeper understanding of the dynamic deficits of individual patients and supplement the clinicians’ decision-making process.

Combing 3D motion capture data with ground reaction force plate data together with musculoskeletal models in so-called inverse workflows allows for the estimation of parameters important for pathology description. These inverse workflows use measured data (i.e., marker trajectories and ground reaction forces) to estimate joint angles and subsequently joint moments using musculoskeletal models. These models are mathematical and physics-based representations of the human body and are used to estimate parameters which cannot be measured such as joint contact loading, and muscle forces. These parameters are often of high clinical interest and already serve to support clinical decision-making in many different clinical use cases (Killen et al., 2020). Related to foot-ankle function, such *in silico* techniques permit to estimate the strain on ligaments and the estimation of muscle forces which could be of particular interest in flatfoot pathologies where ligamentous and muscle dysfunction are often observed (Kaufman et al., 1999; Keegan et al., 2002; Boey et al., 2022). As such the combination of 3D motion capture data, and *in silico* musculoskeletal modelling already provides relevant information on dynamic foot-ankle function potentially prior to, as well as after, insole prescription (Mannisi et al., 2019; Sinclair et al., 2019). However, these approaches do not inform the design process. In fact, the application of the above-mentioned techniques would only be possible if multiple candidate insoles (with different geometries and stiffnesses) were produced, and their effect during dynamic motion subsequently measured and modelled. While this would provide valuable information and potentially the best insole specification, such an approach is infeasible due to increased time as well as cost requirements for manufacturing and testing.

Instead of relying on the manufacturing and testing of multiple insoles, a convenient solution is to instead create an *in silico* representation of the insole which allows for the virtual testing of multiple insole designs without the need to explicitly manufacture them. Previous research has invested in such approaches using highly complex finite-element analysis (Telfer et al., 2014; Telfer et al., 2017). Although these approaches allow integrating highly complex material definitions for the insole as well as the studying the effect on skin tissue pressure, these models often require a specialized and expensive computational infrastructure, infeasible computational times, highly specialized knowledge, and expertise to create and execute, and typically these models do not consider the gait cycle and dynamics of the entire foot-ankle complex.

In this paper we will describe an *in silico* workflow which combines motion capture data, musculoskeletal modelling, and a simplified insole model to predict the effect of a prescribed on a participants’ gait pattern, with specific interest in flatfoot deformity. Such a predictive model would permit the optimization of mechanical parameters for insole design to correct the highly complex and dynamic behavior of the foot-ankle during gait.

## Materials and equipment

The developed workflow was implemented and tested on twenty-seven participants containing a combination of healthy participants (*n* = 7) and patients with flatfoot deformity (*n* = 20). These participants were measured as part of an industry collaboration project (Flemish Government IWT: *Aladyn* project) between KU Leuven and industrial partner Materialise Motion. Each participants’ data was collected as part of an extensive insole testing process whereby multiple insoles and highly detailed foot-ankle motion-capture data were collected in different conditions, including barefoot, in a standardized shod condition (i.e., “labshoe” condition), and with two different types of insoles both designed to apply a mid-forefoot correction.

**FIGURE 1:**
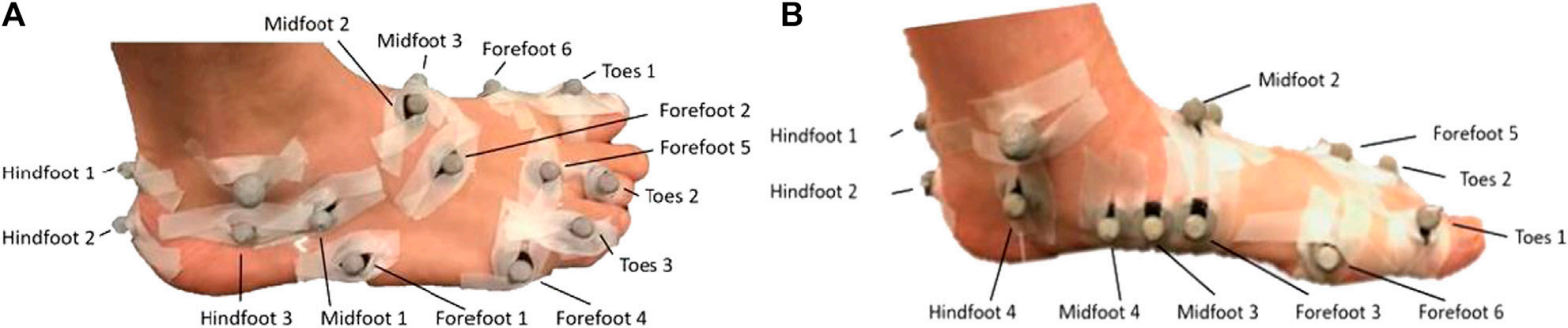
View of the lateral aspect **(A)** and medial aspect **(B)** of a participant fit with the extensive foot-ankle centered marker with markers placed on each of the modelled bone segments.

Participants were fitted with an extensive foot-ankle centered motion capture marker set (Figure 1) with the addition to a four-marker cluster on the tibia. Following fitting of the marker set, participants performed a static calibration trial standing in a pre-defined position (Cappozzo et al., 1995) which was captured by a Vicon (*Vicon*, Oxford Metrics, United Kingdom) motion capture system consisting of 16 cameras collecting at 200 Hz. In addition to marker trajectories, ground reaction forces from in-ground force plates (AMTI, 1,000 Hz) were acquired synchronously. For each participant, and each condition, four repeated walking trials were collected and used in the developed workflow. Marker trajectories were exported from motion capture data following standard labelling and gap filling procedures in Vicon Nexus (*Vicon*, Oxford Metrics, United Kingdom). Measured data was first used to define the initial walking gait pattern using data from the shod condition where each participant wore a standardized lab footwear without insoles.

## Methods

**FIGURE 2:**
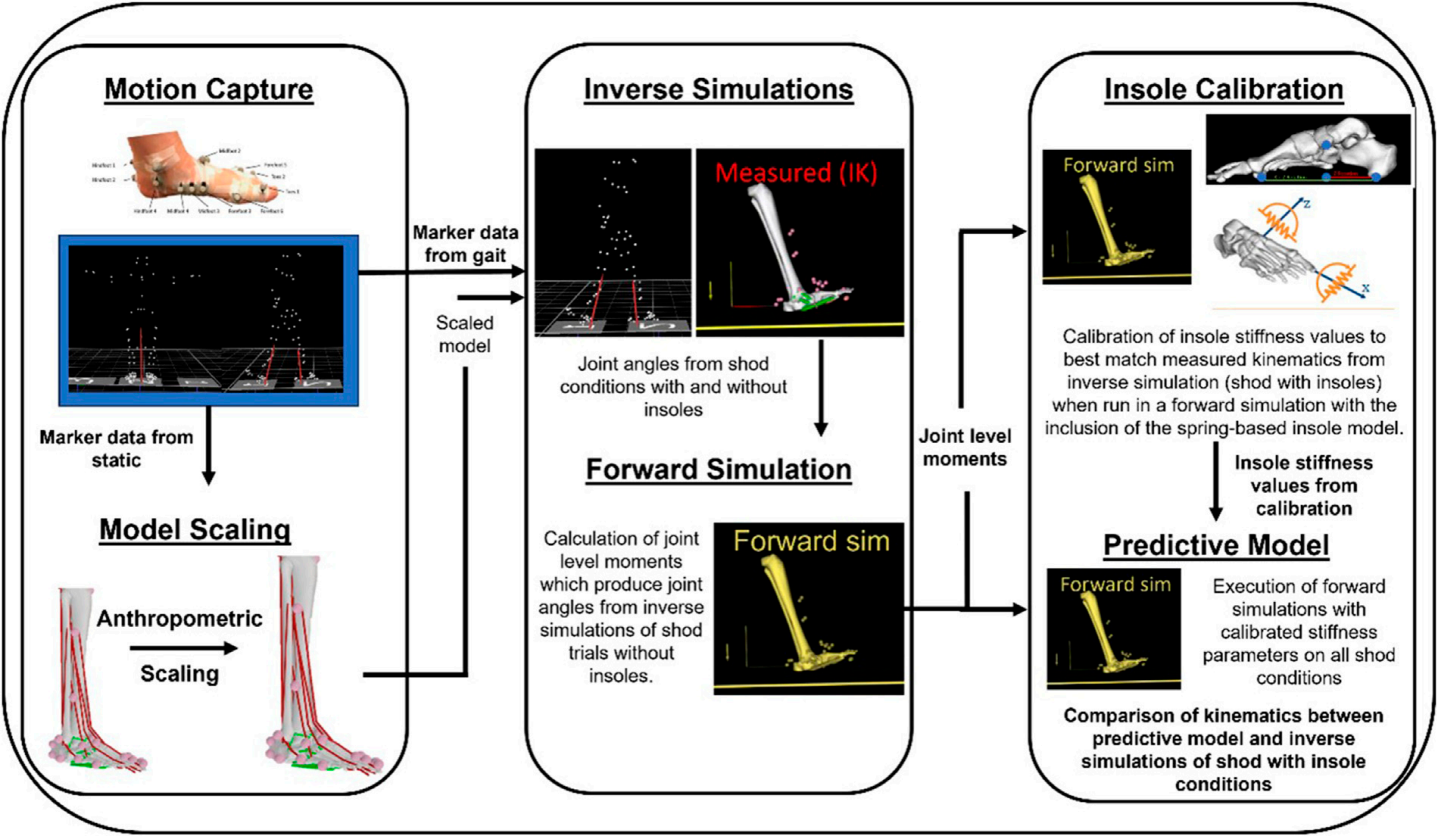
Overview schematic of the workflow from data collection (Motion Capture), musculoskeletal model personalization (Model Scaling), estimation of joint kinematics from each condition (Inverse Simulations), calibration of the insole model stiffness values (Insole Calibration), and the combination of forward simulation outputs (i.e, from shod without insole condition) with the calibrated insole model to estimate individual patient responses (Predictive Model).

The developed *in silico* workflow (Figure 2) is implemented in OpenSim (version 3.3). In short, the workflow combines *in-vivo* measured marker trajectories with an extended musculoskeletal model of the foot-ankle complex to determine patient-specific foot-ankle kinematics and moments. A model of the insole consisting of multiple springs is added to the mid/forefoot segments and a predictive simulation then calculates the estimated change in foot-ankle kinematics. The stiffness of these springs is defined in a calibration step where we iteratively vary spring stiffness constants to best match average measured insole condition. Each of these steps is described in detail in subsequent sections.

### Musculoskeletal model

**FIGURE 3:**
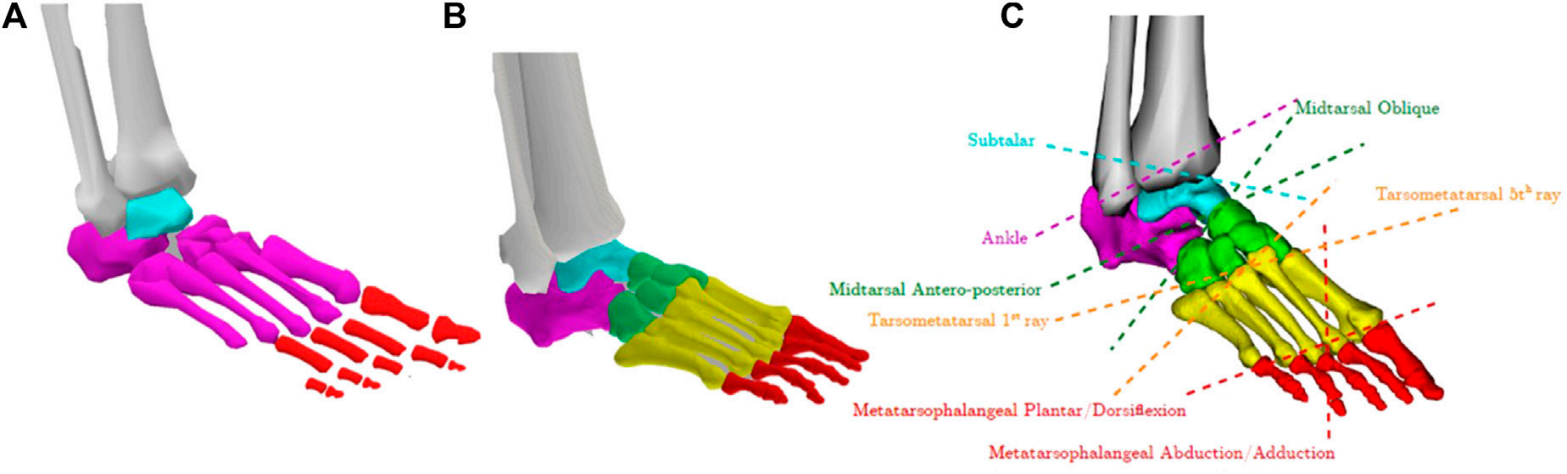
**(A)** Typical representation of a 3 degrees of freedom foot-ankle in many generic musculoskeletal models where each colour represents a different foot segment. **(B)** The previously developed complex foot-ankle musculoskeletal model with **(C)** 8 degree of freedom (Malaquias et al., 2017).

When considering *in silico* applications for insole design, an adequate representation of the complex foot-ankle structure, including different bones, joints and degrees of freedom is vital. Whereas the inclusion of complex hip and knee joint definitions received quite some attention, relatively less attention is paid to include more complex definitions of the ankle and foot joints within musculoskeletal models. Hence, degrees of freedom in the ankle and foot joints are often highly simplified (Figure 3). Specifically, these models often neglect the degrees of freedom of the mid- and forefoot joint, often treating them as a single rigid body. As a result, many musculoskeletal models only have three degrees of freedom, specifically ankle and metatarsal-phalangeal joint plantar-/dorsi-flexion, and subtalar inversion/eversion. While potentially not influential when studying more proximal joints (i.e., knee or hip) these simplifications are a major hurdle when studying the foot and ankle, particular in those with potential foot pathologies relating to structural abnormalities and consequent dysfunction. As such researchers have developed more complex foot-ankle musculoskeletal models which contain more physiological and functionally complete representations (Oosterwaal, 2016; Malaquias et al., 2017; Maharaj et al., 2022).

**FIGURE 4:**
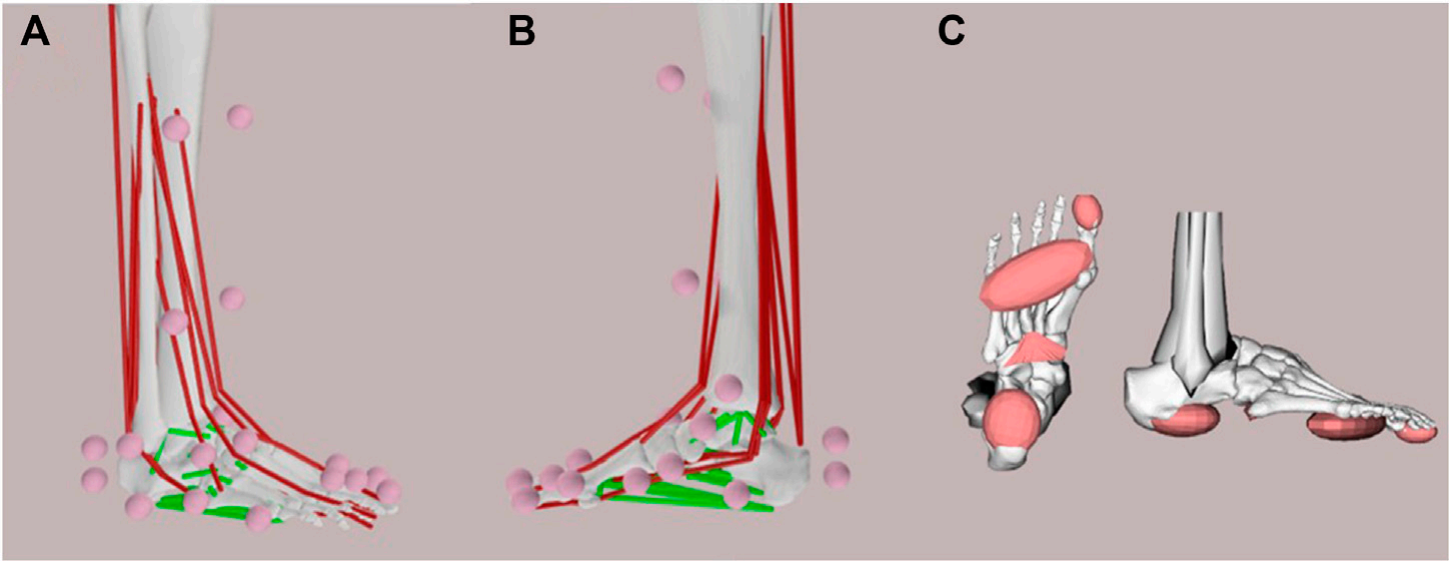
**(A)** Lateral and **(B)** medial view of the extended foot-ankle musculoskeletal model where motion capture markers (pink dots), muscles (red lines), and ligaments (green lines) are visualized. **(C)** Contact surfaces under the foot which model the interaction between the foot and the ground.

Our workflow for insole design integrated a musculoskeletal model developed in our group (Malaquias et al., 2017) that contains 8 degrees of freedom (Figures 3B, C) and a complex representation of the ligaments and muscles of the foot-ankle complex (Figures 4A, B). For use in forward simulations, our model needs to contain a contact model representing the interaction between the foot (our model) and the ground. To do this, we utilized contact ellipsoids attached to specific foot bones (i.e., Calcaneus, midfoot, forefoot, and toes) and an infinite plane for the ground (Figure 4C). Contact geometries were based on medical imaging from 12 individuals where contacting regions of the foot were extracted (Malaquias, 2022). The average shape and size of these geometries were then added to the generic OpenSim model. Contact between these foot geometries and the ground were modelled using an elastic foundation formulation whereby force was proportional to penetration of the foot geometries and the ground where stiffness values were defined based on values from previous work (Malaquias, 2022). These ground-foot contact forces in turn induce rotational joint moments which can in turn affect the estimation of foot joint kinematics when included in forward simulations (see later sections). They -by default-do not affect the inverse simulations performed here which estimate foot-ankle kinematics using only the marker-based trajectories.

This extended generic musculoskeletal model was then used for all subsequent steps within the developed framework. A personalized version of this model was generated for each participant where marker trajectories from the static trial were used in anthropometric scaling. Specifically, markers pairs were defined and the ratio between model (i.e., on the generic model) and measured (i.e., from motion capture) distances were used to determine scale factors. These scale factors were then applied to the corresponding model segment to match the gross anthropometry of each individual participant.

### Inverse simulations

For all acquired gait trials, marker positions were exported as. trc files and used in the open-source OpenSim musculoskeletal modelling platform, to estimate joint angles using a standard Inverse Kinematics (Lu and O’Connor, 1999) approach. Estimated joint angles were restricted to the motion of the tibia (6 degrees of freedom) and the 8 degrees of freedom of the foot-ankle. Gait trials were then limited to only include the stance phase of gait based on measured ground reaction forces and standard thresholding techniques (>10 N).

### Preparatory forward simulations

The next step was to ensure that our preparatory forward simulations can recreate estimated joint kinematics from our inverse simulations detailed above. To achieve this goal, we first estimated the joint moments required to produce the measure joint kinematics (using in-built OpenSim tools for moment-driven froward simulations). These joint level moments were then applied and contact forces/moments generated between the foot and ground, in a forward simulation to verify that measured joint kinematics could be recreated. Once we can successfully reproduce our inverse results in our forward simulations—we can then add our insole model to estimate it is kinematic effect. When comparing forward simulation kinematics—to those from inverse kinematics, the average error across all 8 degrees of freedom of the foot-ankle and all participants was 4.69°.

### Insole model

**FIGURE 5:**
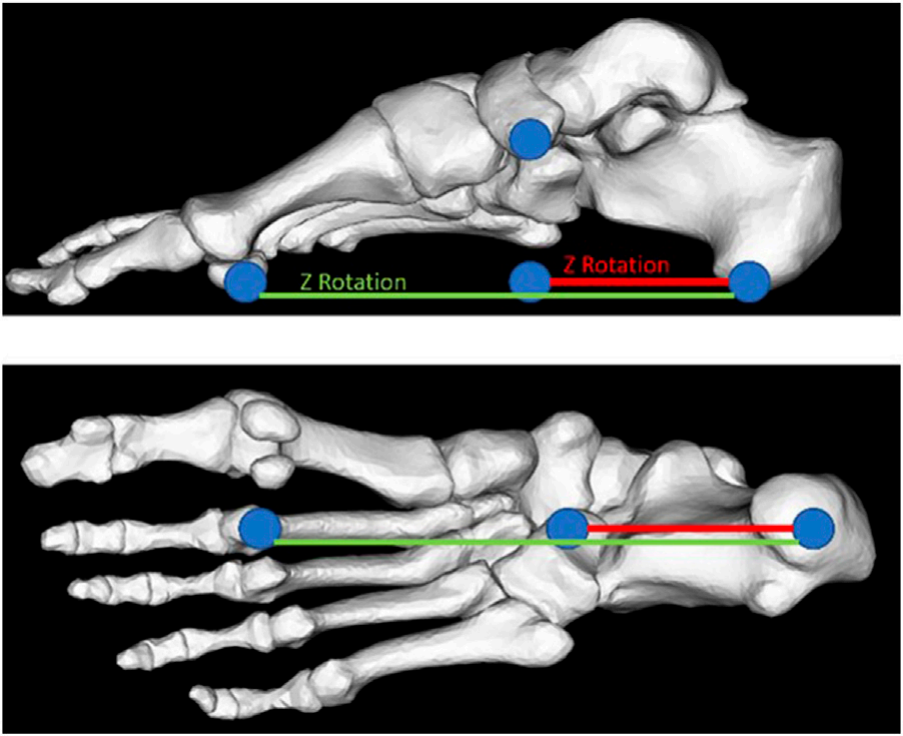
Representation of the implemented spring-based insole model. Shown are the two different bushing forces which are implemented, the first (red) spanning the calcaneus and the midfoot, and the second (green) spanning the calcaneus, midfoot, and metatarsals.

We implemented a lumped insole model in OpenSim utilizing Bushing Forces. These can be thought of as translational or rotational springs which span 2 or more bodies. These springs are characterized by a position (on each body), an initial rotational offset (i.e., orientation) as well as multiple stiffness parameters. These stiffnesses can be applied along any of the three axes and can resist either translations, or rotations around these axes. When two bodies, spanned by a bushing force translate/rotate away from their initial position these springs generate either a force or moment to resist such motion, proportional to their stiffness and the amount of translation/rotation. We reduced the tested insoles to a pair of rotational bushing forces. The first (Figure 5 red line) spanned from the calcaneus to the midfoot which aimed to resist plantar/dorsiflexion of the midfoot relative to the calcaneus. The second spring (Figure 5 green) spans from the calcaneus to the metatarsals and aims to support the medial longitudinal arch of the foot in the distal foot segments.

### Insole calibration

It was our initial intention to estimate the stiffness of each of these springs from mechanical testing of the specific insoles which participants wore. However, as the insole stiffness and geometry parameters (e.g., arch height) were highly variable across individuals, finding a set of stiffness values which reproduced the observed experimental kinematic effect in our simulations was challenging. This was further complicated by the simplified insole definition that does not explicitly account for geometry, and therefore the contact between the foot and the insole, nor the insole/shoe and the ground. Therefore, to derive an initial set of spring stiffness values (for each individual participant) an optimization routine was first executed to calibrate the insole model for each participant.

Insole model calibration was performed for each participant separately. To this end, a single trial (of the participant being calibrated) from the reference shod condition was used as initial input to find a set of insole stiffness parameters (i.e., stiffness and orientation) which reproduces the measured (i.e., from motion capture) response. Specifically, we use the participants’ scaled musculoskeletal model, the joint level moments which produce the kinematics (see Preparatory forward simulations section), and a template insole model (i.e., bushing forces) whose stiffness parameters and orientations will be defined by our calibration process.

Within an optimization routine deployed in Python, we leverage OpenSim API functions to run multiple iterations of forward simulations with differing bushing force (i.e., insole model) parameters. We specifically adjust for both bushing forces, their stiffness (Nm/radian), and orientation. At each iteration the error between predicted (from this specific iteration) and target (average measured insole) kinematics is calculated. Note that despite having gait analysis data throughout the entire stance phase of gait, we focused our error calculations and estimations to the period where the foot was flat on the ground as this is the period where the insole is likely to have the largest effect and the period of interest when assessing corrections. Further, despite estimating a total of 8 degrees of freedom we only consider a subset of foot-ankle kinematics, specifically chopart and tarsometatarsal joint degrees of freedom as these are the joints spanned by our insole model. Each of these joints had two degrees of freedom which were a combination of flexion/extension and inversion/eversion.

**FIGURE 6:**
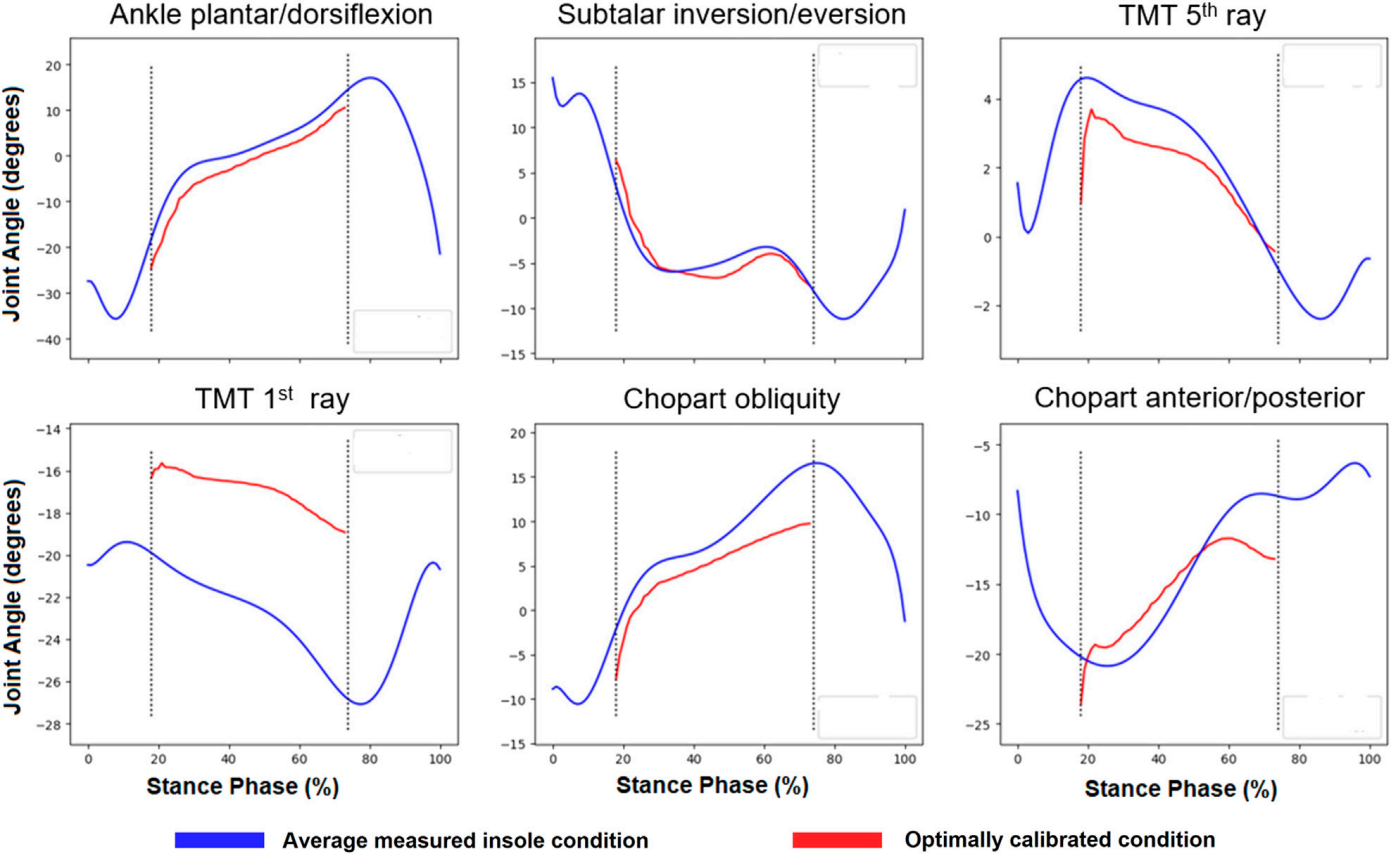
Example of calibration results from a single participant where blue lines indicate the average measured insole condition for selected degrees of freedom from inverse kinematics. Red lines show the kinematics from the optimal calibration solution. Note the dotted black vertical lines indicate where error was calculated within the calibration and correspond to the stage where the foot is flat on the ground.

This calibration is run until the specific stiffness and orientations which yield the minimum error between the two conditions is found—an example of these results for two participants can be seen below (Figure 6). Specifically, the objective function which is evaluated at each iteration calculates the root mean squared error between the average measured insole kinematics and predicted kinematics for each degree of freedom of interest. The average error across the four degrees of freedom, and entire time of interest is then calculated and minimized within the optimization routine. The specific stiffness and orientations yielding the minimum error are then used in the final predictive model and simulations.

### Predictive model

In the final predictive simulation, the participant’s musculoskeletal model, their calibrated insole model (from above) was combined with joint moments (see Preparatory forward simulations section) from the remaining (i.e., excluding the trail used for calibration) reference lab shoe trials to predict a set of simulated insole kinematics across multiple trials. The average simulated insole kinematics were then compared to measured insole kinematics to determine predictive model accuracy. Again, the time of interest was restricted to the period where the foot is flat on the ground, and to the mid- and fore-foot joints.

## Results

**FIGURE 7:**
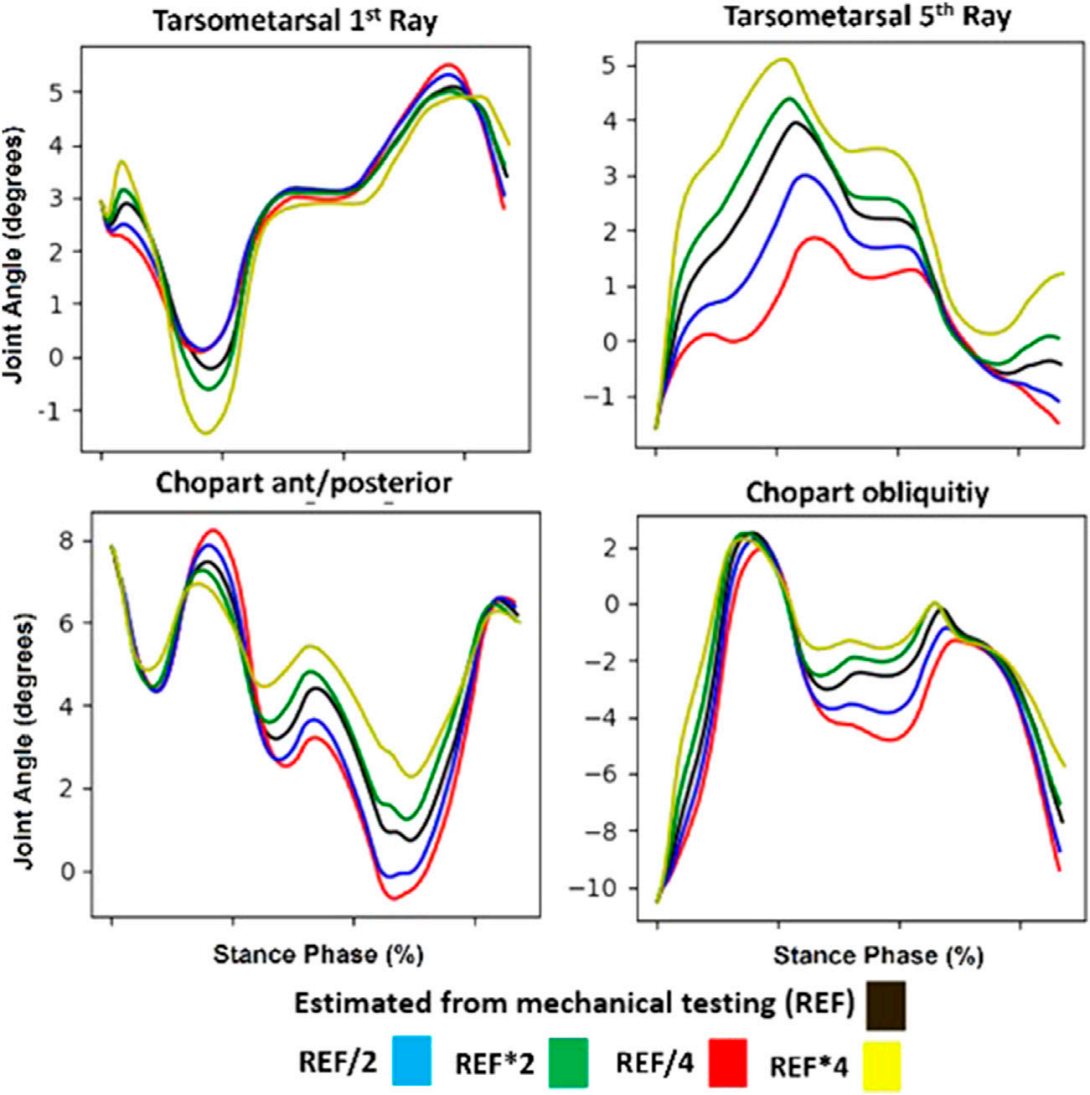
Example simulation results for the targeted mid/forefoot degrees of freedom. Specifically shown are the tarsometatarsal first and fifth ray, and chopart anterior-posterior and obliquity degrees of freedom. Shown in black are forward simulation results with reference insole model stiffness. Shown in Blue and Red are forward simulation results with a half and quarter stiffness compared to the reference respectively. Similarly in Green and Yellow are forward simulation results with double and quadruple stiffness compared to the reference respectively.

When designing this workflow, first, proof of concept simulations were performed to ensure that the implemented insole model which utilizes spring-based bushing forces did alter estimated ankle-foot kinematics, and that these estimations were in fact sensitive to changes in these stiffness parameters (Figure 7). Compared to a reference stiffness as obtained from mechanical testing (black line), increasing or decreasing the insole stiffness effectively alters the estimated kinematics in the targeted degrees of freedom.

**FIGURE 8:**
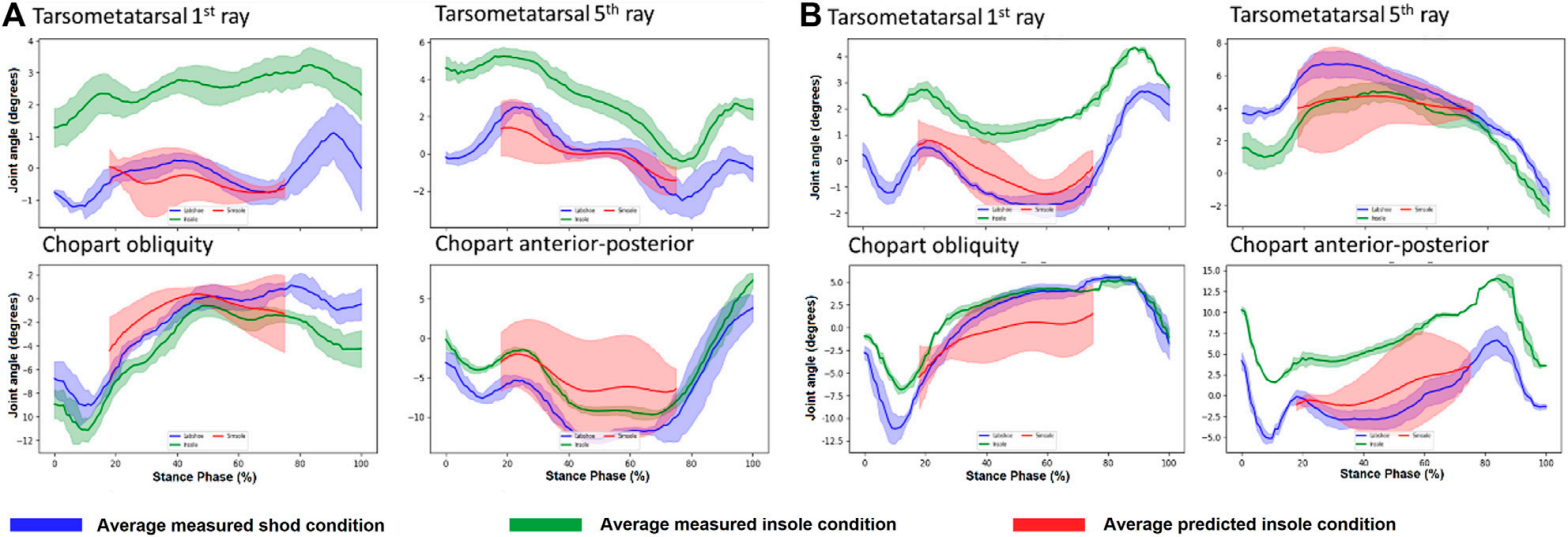
Predicted Insole kinematics (red), measured insole (green), and reference (blue) conditions for two participants **(A, B)**. Note the reason the predicted kinematics are in a smaller range compared to the other conditions is because we focused on a time where the foot was flat on the ground which is representative of this period.

Finally, we used the *in silico* workflow for all participants and the average error between measured and predicted kinematics was calculated. Below (Figure 8) are two exemplary participants for which the measured (shod with insole), and simulated (i.e., predictive insole model) conditions are shown. Across the entire cohort, the average root mean squared error ±standard deviation between measured and simulated kinematics were 4.7 ± 3.1, 4.5 ± 3.1, 2.3 ± 2.3, and 2.3 ± 2.7° for the chopart obliquity, chopart anterior-posterior axis, tarsometatarsal first ray, and tarsometatarsal fifth ray respectively. We observed a wide range of errors in the cohort, with maximum and minimum errors of 0.5–11.7, 1.07–13.12, 0.25–9.31, 0.25–12.5° for the chopart obliquity, chopart anterior-posterior axis, tarsometatarsal first ray, and tarsometatarsal fifth ray respectively.

**FIGURE 9:**
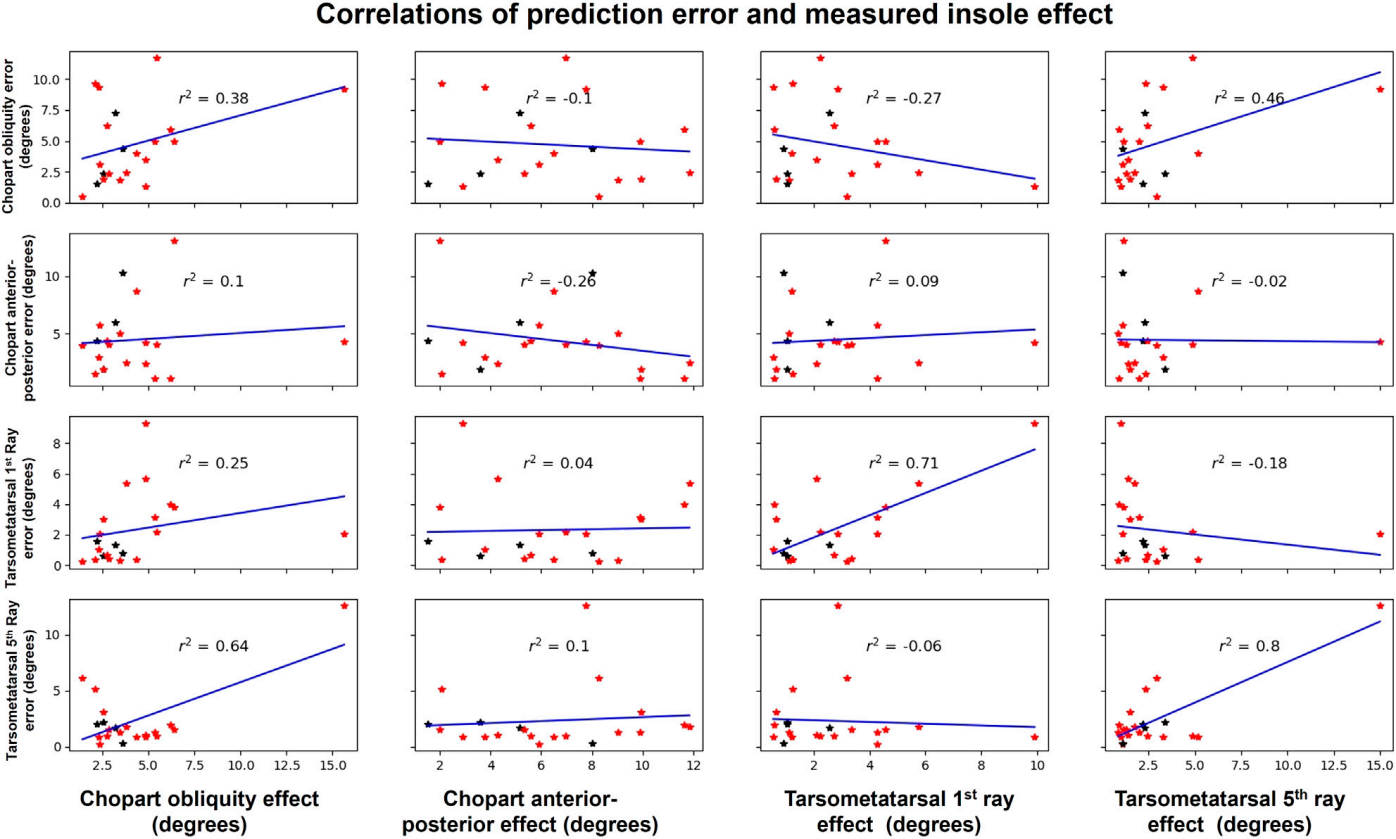
Shown are the measured effect of the insole on X axis for each joint (on a column-by-column basis), and the Y axis showing the prediction error (on a row-by-row basis). Each star represents a different participant were red are flatfeet patients and black are healthy participants. This distinction allowed us to further analyse if the relationship is biased by the population.

Following the analysis of the prediction error of the developed workflow (above), we additionally assessed 1) if the prediction error (i.e., measured vs. predicted) between the joints of interest were correlated, 2) whether the predicted error was correlated with the measured effect (i.e., difference between shod with and without insoles), finally we assessed whether these relationships were different between healthy participants and flatfoot patients. To determine if prediction error was correlated with the measured insole effect (Figure 9), we graphed measured effect on the *X*-axis for each joint (on a column-by-column basis), the *Y*-axis showing the prediction error (i.e., measured vs. predicted) on a row-by-row basis.

Following graphing, a line of best fit was overlayed for each plot and the Pearsons Correlation calculated. In this analysis, we see only one strong correlation (*r* ^2^ 0.8) between the error and effect in the tarsometatarsal fifth ray, however this seems to be biased by an outlier with a large error and large effect (top right). All remaining correlations were weak or negligible except for two moderation correlations between tarsometatarsal fifth ray error and chopart obliquity effect (*r* ^2^ 0.64) as well as chopart obliquity error and tarsometatarsal fifth ray effect (*r* ^2^ 0.46). Examining this relationship in healthy participants (black) and flatfoot patients (red) individually, there appears to be no bias in our workflow between these groups affecting this relationship. The lack of a repeatable relationship suggests that our workflows accuracy is not dependant on a low or high effect, providing confidence in its generalizability.

**FIGURE 10:**
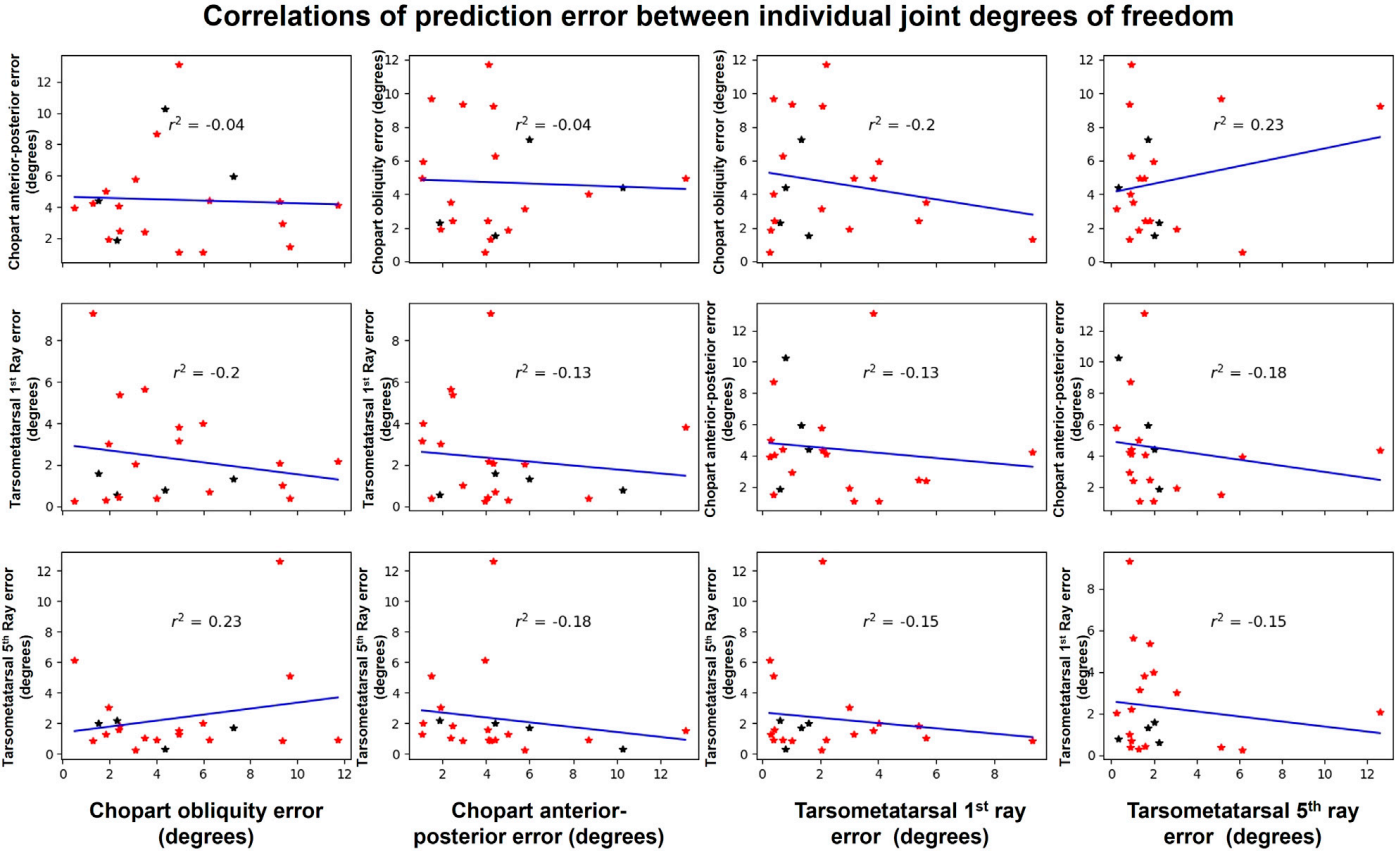
Shown are the errors between the measured and predicted kinematics for all participants for each of the joints of interest. From left to right, on the X axis is the Chopart obliquity, chopart anterior-posterior, tarsometatarsal first ray and finally the tarsometatarsal fifth ray. Each star represents a different participant were red are flatfeet patients and black are healthy participants. This distinction allowed us to further analyse if the relationship is biased by the population.

A similar approach was then applied to determine the correlations between the prediction error (Figure 10) of the different degree of freedom errors (e.g., correlation between chopart obliquity and chopart anterior-posterior). Examining the correlations, only weak and negligible correlations were observed for all comparisons suggesting the error in one degree of freedom in independent of the errors in the remaining degrees of freedom. Similar to the previous comparison, this relationship appears to not be biased or different between the two studied groups.

## Discussion

The design and customization of insoles could benefit from computational approaches that help in determining the appropriate mechanical parameters (i.e., stiffness) to provide the most optimal correction for a targeted foot-ankle kinematic dysfunction. In this paper we aimed to develop a workflow to predict individuals’ response to a specific corrective insole. The workflow described here, implemented in OpenSim, maximally exploits the added value of 3D motion capture, musculoskeletal modeling of the foot-ankle complex, dynamic simulations together with optimization approaches to define the mechanical properties of a lumped insole model that maximally predicts the foot-ankle kinematics.

In contrast to previous work, this workflow was developed using a rigid body musculoskeletal model in OpenSim. Typical insole model-based workflows assessing corrective insole effects utilize finite-element models that explicitly model contact between the foot and the insole to inform in the first instance on predicted changes in plantar contact pressures. Less often the explicit effect on foot-ankle kinematic predictions is targeted. While this approach permits accounting for complex material models relevant for insole design as well as the effective geometrical shape of the insole its drawback is that the time to develop and execute these models is in the magnitude of numerous hours, complicating their use in the context of a parameter optimization approach as described above. Furthermore, it requires highly specific input data for the material parameters that cannot readily be identified in a sole-specific manner, as well as dedicated expertise to build and execute these simulations. The workflow developed here only requires seconds to execute and does not require highly detailed imaging of the participants foot nor the insole. Additionally, the workflow itself is highly automated making execution simple. An additional advantage of this OpenSim musculoskeletal modelling approach is that an entire stance phase and numerous gait cycles can be simulated meaning a larger number of trials can be collected, potentially in different environments and speeds meaning the effect of an insole in variable gait conditions can be tested. This contrasts with previously mentioned finite-element modelling approaches that typically only consider a combination of static or quasi-static time points.

The developed musculoskeletal modeling workflow uses measured motion capture data from shod conditions without insoles as a reference condition and predicts the effect of the insole on foot-ankle kinematics during stance phase of gait using a spring-based insole model. Insole models were calibrated based on measured data to improve the predictive ability of the framework. This was a necessary step as initial parameters derived from mechanical testing did not generate a sufficient effect of the predicted kinematics and often yielded the same kinematics as the reference condition. Although a hurdle of the workflow being fully predictive, this is currently required as there is currently no direct way to translate the properties of corrective insoles from the manufacturer to our musculoskeletal modelling framework. However this is a preliminary implementation which we believe can be phased out in later and more developed implementations when a larger cohort can be modelled and considered.

When considering the magnitude of errors between measured and predicted insole conditions (See Results and Figure 8), these are between 2 and 5° for the tarsometatarsal and chopart joints, respectively. These errors also need to be considered in the context of the actual effect of the insole. The effect of the insole to measured kinematics in many cases was highly variable between participants and in many cases is in the range of error in our predictive model. When examining the average effect of the insole in the measured values we see the following: 4.18 ± 2.98, 6.28 ± 3.11, 2.67 ± 2.23, and 2.75 ± 3.05° for the chopart obliquity, chopart anterior-posterior axis, tarsometatarsal first ray, and tarsometatarsal fifth ray respectively. Importantly, the secondary analysis performed shows our model’s accuracy is not dependent on the magnitude of the response, nor does it seem to be influenced if it is applied to a healthy participant or flatfoot patient providing confidence in the generalizability of the results.

As with any study, several limitations need to be considered. First, the predictive framework is currently reliant of high-fidelity motion capture data which may limit the scope and application of the current approach. Such equipment is not typically available in clinical practice where such an approach would have the most impact. Second, the use of the calibration steps relies on *a-prior* knowledge of an individual’s response which would still require fabrication of numerous insole candidates meaning the framework cannot be run fully predictive.

Future research should focus on reducing the reliance on the use of motion capture data. The use of such data reduces the wider adoption of these approaches as they do not have access to such specialized infrastructure. As this approach is only currently reliant on accurate estimations of joint kinematics, leveraging of other modalities such as video-based analysis or machine learning may provide a viable alternative to inform such a developed workflow in the future. To this end, current low fidelity approaches such as the use of plantar pressure measurements are becoming more common. Such approaches would allow for the application and translation of such a framework in clinics to facilitate its translation and impact on patients. Future research should focus on improving the simplified spring-based insole model by processing larger data sets with a larger variety of insole designs and mechanical properties potentially augmented and assisted by formal mechanical testing procedures. The improvement of this spring-based model will improve the predictive accuracy of the framework. Following these improvements and refinements, a complete clinical validation including not only quantitative measures of kinematic response, but also patients’ subjective outcomes, and clinical outcomes should be done to compare the effectiveness of the *in silico* based workflow for insole design compared to current practice.

This framework serves as an important first step in realizing such an approach not reliant on finite element methods. Examining the results of calibration (Figure 6) using our approach, it is possible to reproduce measured insole gait kinematics using measured shod data (without an insole) and a spring-based insole model. This is a promising outcome as it means that if we can effectively and accurately translate insole design parameters from the manufacturer (e.g., arch support and height, regional stiffness) to spring-based parameters (i.e., bushing force stiffness) the accuracy of this workflow can likely be increased.

## Conclusion

The developed workflow provides a solid basis for future *in silico* work to improve the predictive accuracy of the effect of insoles on foot-ankle kinematics, given that the lumped insole model can be further optimized, in terms of mapping the experimental insole mechanical properties and the formulation of the spring-based constants. Combined, a reduced burden of data collection, and improved spring-based model provides an exciting potential for a fully predictive model to assist design and prescription of corrective insoles and improved patient outcomes.

## Data Availability

The datasets presented in this article are not readily available because Data collected in this research were collected by a commercial company who retain the rights to this data. Requests to access the datasets should be directed to BK bryce.killen@kuleuven.be.
